# Elbow Contracture Secondary to Congenital Shoulder Luxation in a Dog: Surgical Management with Elbow Muscle Release and Circular Osteotomy-Based Shoulder Arthrodesis

**DOI:** 10.3390/ani15182717

**Published:** 2025-09-16

**Authors:** Changhun Ryu, Haebeom Lee, Youngjin Jeon, Jaemin Jeong, Jongpil Yoon

**Affiliations:** Department of Veterinary Surgery, College of Veterinary Medicine, Chungnam National University, 99, Daehak-ro, Yuseong-gu, Daejeon 34134, Republic of Korea; modd3@naver.com (C.R.); seatiger76@cnu.ac.kr (H.L.); orangee0115@gmail.com (Y.J.); klmie800@cnu.ac.kr (J.J.)

**Keywords:** elbow contracture, congenital shoulder luxation, circular osteotomy, shoulder arthrodesis, dog

## Abstract

A 10-month-old Poodle was presented with intermittent non-weight-bearing lameness of the left thoracic limb. Orthopedic and radiographic examinations revealed medial shoulder luxation and markedly reduced elbow extension. A two-stage surgical approach was performed. In the first stage, selective myotomy of periarticular structures, including the biceps brachii–brachialis complex and the extensor carpi radialis muscle, was conducted via medial and lateral approaches. A trans-articular external skeletal fixator was applied to maintain elbow extension. Elbow extension improved from 105° preoperatively to 142°. After confirming functional recovery of the elbow joint, the second stage involved shoulder arthrodesis using a circular osteotomy technique with a radial saw, which enabled fine-tuned intraoperative adjustment of limb alignment based on the contralateral limb posture. At nine months postoperatively, the patient exhibited a symmetrical gait, full weight-bearing, and no evidence of discomfort on range of motion assessment. This case highlights the clinical relevance of secondary elbow contracture associated with congenital shoulder instability and suggests that a combination of targeted muscle release and adjustable arthrodesis may offer favorable outcomes in managing complex joint dysfunction.

## Simple Summary

Elbow joint contracture can significantly impair thoracic limb function in dogs, particularly due to the joint’s critical role in weight-bearing and extension. While most contractures result from trauma or neuromuscular conditions, this case demonstrates that chronic disuse from congenital shoulder luxation may also lead to secondary elbow contracture. A 10-month-old presented with clinical signs of limited elbow extension and medial shoulder luxation. Surgical intervention was performed in two stages: first, selective release of periarticular muscles improved elbow range of motion; second, shoulder arthrodesis using a circular osteotomy allowed accurate limb alignment. The dog recovered full limb function postoperatively. This report emphasizes the importance of evaluating adjacent joints in case of chronic joint instability. Early identification of secondary changes in related joints can support more effective treatment planning and improve long-term outcomes.

## 1. Introduction

Elbow joint contracture, although relatively uncommon in small animals, is a clinically significant condition that can severely limit range of motion and result in gait dysfunction [1,2].

It typically arises secondary to trauma, neuromuscular disorders, or chronic disuse [2,3,4,5]. Given the elbow joint’s essential role in weight-bearing and limb extension during locomotion [1], limited mobility at this joint can substantially affect overall limb function.

One potential but under-recognized cause of chronic thoracic limb disuse is congenital shoulder luxation, an orthopedic condition in dogs characterized by glenoid dysplasia and glenohumeral joint instability [6,7]. Affected animals often demonstrate impaired use of the thoracic limb from an early age, predisposing them to articular cartilage degeneration and periarticular soft tissue fibrosis secondary to prolonged disuse [4]. Over time, reduced limb loading may lead to adaptive shortening and fibrosis of periarticular soft tissues in distal joints [5], including the elbow. However, to the authors’ knowledge, secondary elbow joint contracture associated with congenital shoulder luxation has not been previously reported in veterinary literature, despite this plausible pathomechanism.

Management of elbow joint contracture is rarely reported in veterinary medicine and is typically limited to physiotherapy [2]. Surgical treatment, especially for chronic cases, remains poorly described. In contrast, human medicine often employs surgical procedures, such as biceps tendon release, capsulectomy, or ligamentous release, for chronic or refractory cases [8].

Surgical correction of congenital shoulder luxation often necessitates arthrodesis, particularly in cases with chronic instability and glenoid dysplasia [6,9]. A major technical challenge in shoulder arthrodesis is achieving proper limb alignment at a functional angle [9]. Conventional planar osteotomies offer limited intraoperative flexibility for adjusting the fixation angle. In contrast, circular osteotomy, previously reported in stifle arthrodesis [10], allows fine-tuned angular correction during fixation and may address this limitation. Based on these advantages, circular osteotomy was applied in the present case to facilitate precise angular modulation during shoulder arthrodesis.

This case report describes a dog presenting with severe elbow contracture secondary to chronic congenital shoulder luxation. The case details a two-stage surgical strategy involving selective muscle release and shoulder arthrodesis using circular osteotomy to restore functional limb alignment.

## 2. Case Description

This case report was prepared in accordance with the CARE guidelines. A 10-month-old spayed female Poodle was presented for evaluation of intermittent non-weight-bearing lameness of the left thoracic limb. According to the owner, the lameness had been intermittently observed since the dog was adopted at three months of age, with gradual worsening of clinical signs over time. At that time, medial shoulder luxation was diagnosed at a local veterinary clinic, while no abnormalities were detected in other joints, and no treatment was initiated.

On gross gait analysis at the authors’ institution, intermittent non-weight-bearing lameness of the left thoracic limb was observed. Orthopedic examination revealed a medial luxated left shoulder and restricted passive range of motion (ROM) in the left elbow. Passive ROM testing under sedation elicited a capsular and soft end feel during extension. Under sedation, extension and flexion angles of the right elbow measured 145° and 20°, respectively, whereas those of the left elbow were 105° and 22° (Figure 2A).

**Figure 1:**
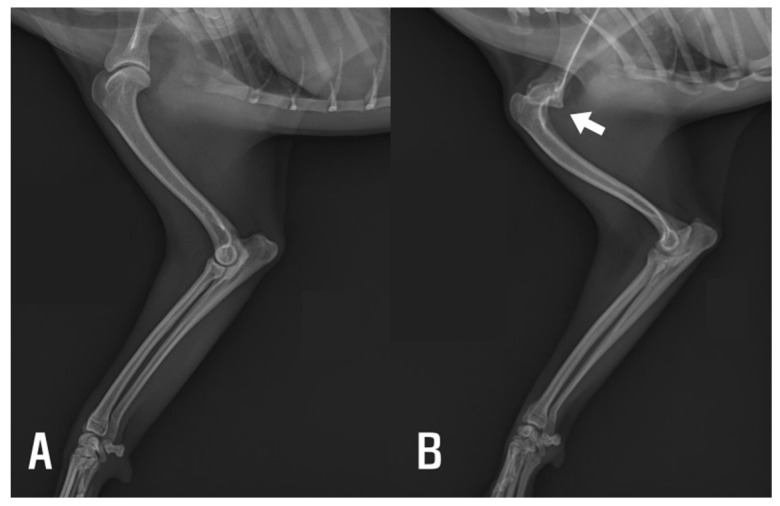
(**A**) Right forelimb showing normal glenohumeral joint alignment. (**B**) Left (affected) forelimb demonstrating displacement of the humeral head consistent with flattened glenoid cavity (arrow).

Radiographic evaluation revealed dysplasia of the left scapular glenoid cavity and humeral head, supporting a diagnosis of congenital shoulder luxation (Figure 1). Advanced imaging, such as CT or MRI, was not performed due to the owner’s decision; however, given the absence of elbow osseous abnormalities on radiographs, the restricted ROM observed clinically was considered consistent with periarticular soft tissue contracture.

In the two weeks prior to surgery, the owner performed passive range of motion exercises of the elbow three times daily. However, no clinical improvement was observed, and the elbow angle remained unchanged.

Surgical treatment was performed in two stages, with initial correction of the elbow joint contracture, because simultaneous application of a plate for shoulder arthrodesis and a temporary trans-articular ESF for the elbow was considered technically difficult.

Both surgical procedures were performed under the same anesthetic protocol. Anesthesia was initiated with intravenous administration of midazolam (0.2 mg/kg, IV) as premedication. Induction was achieved using propofol (6 mg/kg, IV), and anesthesia was maintained with inhaled isoflurane delivered in oxygen. Remifentanil was administered as a continuous infusion (0.1–0.3 μg/kg/min) for intraoperative analgesia. Prophylactic antimicrobial coverage was achieved with intravenous cefazolin (22 mg/kg), administered 30 min prior to skin incision and once more intraoperatively, as the procedure lasted approximately 90 min.

**Figure 2:**
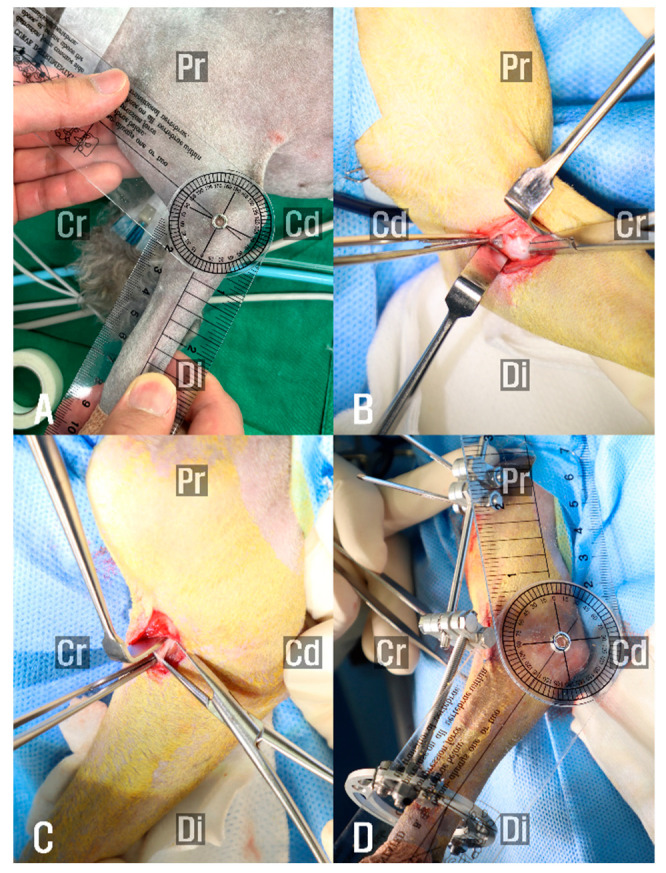
(**A**) Preoperative photograph of the left elbow showing a restricted extension angle of approximately 105°. (**B**) Medial approach showing release of the biceps brachii and brachialis complex insertion site. (**C**) Lateral approach showing release of the extensor carpi radialis muscle insertion site. (**D**) Application of trans-articular ESF following muscle releasing. Elbow stabilized at approximately 135° of extension, demonstrating improved range of motion compared to the preoperative state. Cr, cranial; Cd, cauda; Pr, proximal; Di, distal.

The patient was positioned in dorsal recumbency to allow simultaneous access to both the medial and lateral aspects of the elbow [11]. A medial skin incision was made to evaluate the extent of joint capsular contracture. Soft tissue structures, including the subcutaneous tissue and antebrachial fascia that were under tension during elbow extension, were selectively released by sharp dissection of the fascia followed by blunt dissection. The insertion site of the biceps brachii–brachialis muscle complex was then carefully separated and partially dissected to facilitate release of the contracted soft tissue (Figure 2B). Although this intervention led to an improvement in the extension angle, the achieved range was deemed insufficient for functional recovery, and assessment was based on intraoperative manual evaluation of elbow joint motion rather than objective angle measurement.

**Figure 3:**
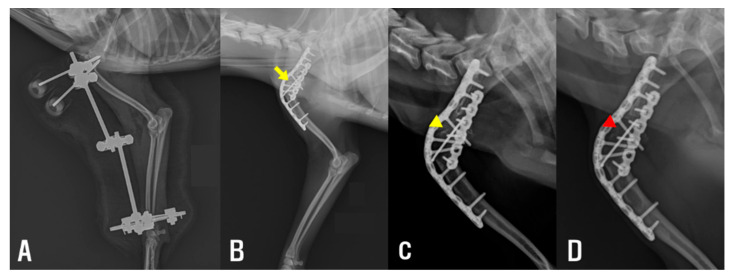
(**A**) Immediately after elbow muscle release, showing improved elbow extension and joint alignment. (**B**) Immediately after shoulder arthrodesis, demonstrating appropriate implant placement. A gap is observed at the arthrodesis site (yellow arrow). (**C**) One month postoperatively after shoulder arthrodesis, showing a reduction of the gap at the arthrodesis site compared with the immediate postoperative image (yellow arrowhead). (**D**) Four months postoperatively after shoulder arthrodesis, showing near-complete disappearance of the gap at the arthrodesis (red arrowhead).

Subsequently, a lateral approach was performed to further release periarticular soft tissues. The insertion site of the extensor carpi radialis muscle was also released (Figure 2C), resulting in a measurable improvement in the elbow’s ROM, with the range of motion extending to approximately 135°. To maintain the corrected extension angle and prevent recurrence of contracture, a trans-articular ESF was applied (Figure 2D). A 35 mm circular ring (IMEX Veterinary, Longview, TX, USA) was affixed to the distal radius using two 0.9 mm Kirschner wires (K-wire, Top Medical Company, Seoul, Republic of Korea). Additionally, two 2.4 mm end-threaded pins (Duraface ESF, IMEX Veterinary) were placed in the humerus and secured using lateral connecting rods. Postoperatively, the patient received cefazolin (22 mg/kg, intravenously) for seven days until negative culture results were confirmed. Remifentanil (0.1 μg/kg/min, constant rate infusion) was tapered over two days. Meloxicam was administered (0.2 mg/kg, subcutaneously) on day 1, followed by oral administration (0.1 mg/kg) once daily for an additional 6 days. The patient was discharged one week after surgery and continued with outpatient management consisting of wound care. The fixator was maintained for two weeks (Figure 3A). Passive ROM exercises were initiated immediately following fixator removal to promote soft tissue flexibility and to reduce the risk of recurrence. These exercises were performed three times daily on both the elbow and carpus within a pain-free range of motion. Gradual improvement in elbow extension was observed over the following weeks. The frequency of intermittent non-weight-bearing episodes appeared to decrease over time, based on subjective owner observation and clinical examination. Prior to shoulder arthrodesis, elbow ROM measurements demonstrated substantial recovery, with the right elbow showing flexion of 20° and extension of 148°, and the left elbow showing flexion of 22° and extension of 142°.

One month after the initial surgery, definitive surgical stabilization of the left shoulder was performed. Lateral recumbency was used, and the shoulder joint was exposed via a craniolateral approach, as previously described [9]. Due to the presence of glenoid dysplasia and chronic luxation, anatomical reduction was not feasible; therefore, shoulder arthrodesis was performed as a salvage procedure.

**Figure 4:**
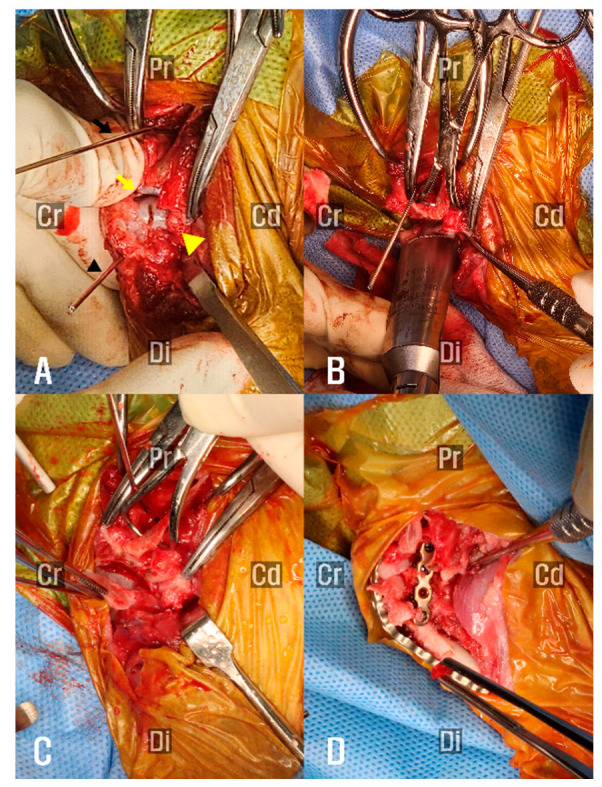
(**A**) Left shoulder joint exposed via a craniolateral approach. A 1.6 mm K-wire was inserted into the humerus perpendicular to its anatomical axis (yellow arrowhead), and a second K-wire was placed in the scapula (yellow arrow) in the same manner. These pins served as visual guides to assist in maintaining proper alignment during the osteotomy. The glenoid cavity of the scapula is indicated by the yellow arrow, and the humeral head is indicated by the yellow arrowhead. (**B**) Circular osteotomy of the articular surfaces performed using a radial saw. (**C**) Articular surfaces removed, and concave–convex interface prepared for fusion. (**D**) Shoulder joint stabilized with a bone plate following alignment and compression of the osteotomized surfaces. Cr, cranial; Cd, cauda; Pr, proximal; Di, distal.

To guide the osteotomy planes and ensure accurate alignment, a 1.6 mm K-wire was inserted perpendicular to the anatomical axis of the scapula, and a second K-wire was inserted perpendicular to the axis of the humerus (Figure 4A). These pins served as independent visual references to maintain proper orientation during circular osteotomy and minimize the risk of angular deformity.

A circular osteotomy was performed at the scapular glenoid cavity and the humeral head, including the greater tubercle using a 12 mm radial saw (Synthes, Oberdorf, Switzerland; Figure 4B,C), to allow controlled adjustment of the limb alignment angle. The joint was then stabilized at an angle of approximately 120°, a position considered appropriate for maintaining functional limb posture during ambulation. Internal fixation was achieved using a combination of a K-wire and two contoured reconstruction locking plates. A single 1.6 mm K-wire was inserted from the greater tubercle of the humerus into the scapular body to maintain the reduction of the osteotomy surfaces and augment stabilization of the bones across the fusion site. Subsequently, internal fixation was completed with anatomically contoured locking plates. A 1.5 mm/2.0 mm poly-axial locking plate (Arix Vet, Jeil Medical Corp., Seoul, Republic of Korea) was contoured and applied to the craniolateral aspect of the scapula and proximal humerus, with screws directed to engage both the scapular spine and body. An additional 1.2 mm locking plate (Arix 1.2; Arix vet, Jeil Medical Corp.) was contoured and applied to the caudolateral aspect of the humerus to augment construct rigidity (Figure 4D).

The same postoperative analgesic and antimicrobial protocols used in the initial surgery were applied following the second procedure. The patient began controlled leash walking the day after surgery, limited to approximately 5 min per session, three times daily, for elimination purposes, and showed progressive improvement in limb function. Passive ROM exercises were performed in the same manner as after the initial surgery. In addition, laser therapy was administered during the one-week hospitalization period at a dose of 4–6 J/Spot over the scapular region and elbow. The patient was discharged one week postoperatively with instructions for activity restriction at home, along with continuation of passive ROM exercises for both the elbow and carpus. Partial weight-bearing was observed during the early postoperative period, with gradual progression to full ambulation at the one- and four-month re-evaluations, during which follow-up radiographs confirmed appropriate maintenance of the implant and progressive healing at the fusion site (Figure 3C,D). At the nine-month follow-up, the elbow joint maintained an extension angle of approximately 140°, with no evidence of pain or discomfort on passive range of motion assessment. The dog displayed a symmetrical gait and full functional recovery based on visual examination.

## 3. Discussion

This case describes the successful two-stage surgical management of a dog presenting with congenital shoulder luxation complicated by secondary elbow joint contracture. To the authors’ knowledge, this concurrent presentation has not been previously reported in the veterinary literature. The case underscores the importance of thorough evaluation of adjacent joints in patients presenting with chronic thoracic limb dysfunction. In this patient, functional limb recovery and long-term gait normalization were achieved through a staged surgical approach consisting of initial correction of the elbow joint contracture followed by shoulder arthrodesis.

Joint contracture may arise from pathological changes in periarticular soft tissues [12]. Chronic disuse or altered biomechanics can lead to fibrotic changes within these structures, ultimately restricting joint mobility [4].

Elbow joint contracture, in particular, is rarely reported in small animals but may cause substantial gait dysfunction due to the elbow’s critical role in weight-bearing and forelimb extension [2]. In the present case, the contracture was presumed to be secondary to disuse resulting from early-onset shoulder instability. Additionally, chronic glenohumeral malalignment may have altered the mechanical loading and muscular tension pattern across the limb, potentially contributing to adaptive shortening or fibrosis of periarticular structures around the elbow.

Although elbow flexion was relatively preserved, extension was markedly restricted, suggesting chronic fibrosis of extensor structures. Under normal conditions, the canine elbow joint moves through a range of approximately 48.1° to 70° during ambulation to accommodate normal gait mechanics [1]. In this case, the restricted extension range was insufficient to meet this functional requirement, resulting in intermittent non-weight-bearing gait during ambulation. Given their anatomical roles in elbow mechanics, the biceps brachii–brachialis complex and extensor carpi radialis were suspected as primary contributors to the contracture [13]. The biceps brachii–brachialis complex, which inserts on the proximal ulnar tuberosity [14], acts as a powerful elbow flexor and may significantly resist extension when shortened or fibrotic. Similarly, the extensor carpi radialis, although primarily responsible for carpal extension, contributes to elbow positioning through fascial continuity [13].

In human medicine, soft-tissue-related elbow contractures have been managed with conservative approaches, such as splinting and rehabilitation [8,15]. However, these approaches often require prolonged treatment durations and specialized equipment [15], which may not be feasible or practical in veterinary patients [16].

In the present case, a surgical approach focusing on selective soft tissue release resulted in substantial improvement in elbow extension without the need for joint capsulectomy or osseous intervention [17,18]. These findings indicate that the contracture was primarily extrinsic to the joint, with muscular and fascial structures serving as the principal contributors to the observed functional limitation.

The recurrence of joint contracture has been reported in up to 37% of cases in human medicine [8]. Based on this, and to minimize the risk of postoperative fibrosis or additional elbow contracture following surgery [2,3], trans-articular ESF was applied to maintain elbow extension and prevent soft tissue re-tightening prior to shoulder arthrodesis. In the present case, the trans-articular ESF was maintained for two weeks. This application period was intended to address residual medial shoulder luxation and to prevent disuse associated with postoperative pain, while avoiding prolonged elbow immobilization that could further reduce the range of motion [19]. Temporary stabilization with a trans-articular ESF was, therefore, employed, followed by definitive shoulder arthrodesis. A two-week interval was selected to allow early elbow rehabilitation and to ensure the absence of infection, thereby providing a safer environment for subsequent internal fixation. Nevertheless, the use of ESF carries inherent limitations. The most common complication is pin tract infection [20], which fortunately did not occur in the present case and allowed timely progression to shoulder arthrodesis. In addition, patient compliance plays a critical role in the success of ESF management; in the present case, favorable tolerance and cooperation facilitated the safe application of this technique.

Notably, previous studies have demonstrated that transection of the brachii–brachialis complex, commonly performed during surgical treatment for medial compartment disease of the elbow [2,21], does not result in significant postoperative functional impairment. Similarly, although extensor carpi radialis release is not yet standardized in veterinary orthopedic practice, it is an established technique in the management of elbow contracture in human medicine [17]. In dogs, partial resection of the extensor carpi radialis muscle has also been reported without subsequent functional deficits [18]. These findings support the safety and clinical utility of selectively releasing these structures when indicated.

In the present case, definitive management of congenital shoulder luxation was achieved through shoulder arthrodesis, thereby eliminating the primary cause of chronic thoracic limb dysfunction. Shoulder arthrodesis remains the most commonly employed surgical treatment for congenital shoulder luxation, with successful outcomes dependent on thorough articular cartilage removal and achieving fusion at a functional standing angle [9]. To accomplish this, circular osteotomy was selected because it allows intraoperative adjustment of the fixation angle, particularly in the sagittal plane, enabling precise control of humeral flexion and extension relative to the scapula. This technique, previously described in stifle arthrodesis [10], offers superior versatility in achieving functional limb alignment compared with conventional planar osteotomies. However, if the osteotomy plane is not oriented perpendicular to both the craniocaudal and proximodistal axes of the limb, there is a risk of inducing angular deformities during fixation [22]. To mitigate this risk and ensure accurate osteotomy orientation, a 1.6 mm K-wire was inserted into both the scapula and humerus, each perpendicular to its respective anatomical axis, serving as a visual alignment guide throughout the osteotomy procedure.

When planning the osteotomy, the size of the radial saw was determined to encompass the entire glenoid cavity while preserving bone stock and avoiding extension to the level of the suprascapular nerve. On humeral side, the same saw diameter was applied to ensure balanced osteotomy preparation. Because the humeral head was smaller than the glenoid cavity in the present case, the osteotomy was extended to include the greater tubercle, thereby maximizing the contact surface for bone fusion.

This report has several limitations. First, as a single case report, the findings cannot be generalized without further supporting evidence. Second, there was no direct comparison with patient-specific conservative or rehabilitation management strategies, which limits the contextualization of the surgical outcome. Third, advanced imaging, such as MRI, was not performed to evaluate periarticular soft tissue involvement. Fourth, histopathological examination was not obtained, which could have provided more objective confirmation of the underlying pathology. Finally, long-term follow-up is required to further validate the durability of the clinical outcome.

## 4. Conclusions

In conclusion, this case highlights the potential for secondary elbow joint contracture in dogs with congenital shoulder luxation. Chronic disuse resulting from shoulder joint malalignment may restrict mobility in adjacent joints, even in the absence of primary pathology. Thorough assessment of overall limb function, including elbow joint mobility, is, therefore, essential in these patients.

When functional limitations are identified, early surgical intervention, such as targeted release of periarticular musculature at the elbow, may be necessary to restore limb use and optimize conditions for definitive procedures, such as shoulder arthrodesis. In the present case, this approach combined with circular osteotomy-based shoulder fusion resulted in successful restoration of functional gait. These findings support the use of a staged surgical strategy and emphasize the importance of comprehensive limb evaluation in complex congenital orthopedic conditions.

## Data Availability

The original contributions presented in the study are included in the article/Supplementary Materials. Further inquiries can be directed to the corresponding author.

## Supplementary Materials

The following supporting information can be downloaded at: https://www.mdpi.com/article/10.3390/ani15182717/s1. Video S1: Preoperative slow-motion video demonstrating intermittent non-weight-bearing gait. Video S2: Postoperative gait: A normal gait was confirmed up to 9 months postoperatively. Care checklist: The completed CARE checklist for this case report is available online as supplementary material.
