# Supplementary material for: Elbow Contracture Secondary to Congenital Shoulder Luxation in a Dog: Surgical Management with Elbow Muscle Release and Circular Osteotomy-Based Shoulder Arthrodesis
Source: Animals (Basel). 2025 Sep 16;15(18):2717. doi: 10.3390/ani15182717 (PMC12466446; doi:10.3390/ani15182717)
Supplement: Supplementary file 1 [file animals-15-02717-s001.zip › CARE-checklist.pdf]

CARE case report guidelines

CARE Checklist of information to include when writing a case report

(cc) BY-NC-ND

Topic	Item	Checklist item description	Reported on Line
**Title**	1	The diagnosis or intervention of primary focus followed by the words "case report"	Line 1,Line 2-4
**Key Words**	2	2 to 5 key words that identify diagnoses or interventions in this case report, including "case report"	Line 40-41
**Abstract (no references)**	3a	Introduction: What is unique about this case and what does it add to the scientific literature?	Line 36-39
	3b	Main symptoms and/or important clinical findings	Line 24
	3c	The main diagnoses, therapeutic interventions, and outcomes	Line 25-35
	3d	Conclusion—What is the main "take-away" lesson(s) from this case?	Line 36-39
**Introduction**	4	One or two paragraphs summarizing why this case is unique (**may include references**)	Line 72-75
**Patient Information**	5a	De-identified patient specific information.	Line 77-78
	5b	Primary concerns and symptoms of the patient.	Line 78-79
	5c	Medical, family, and psycho-social history including relevant genetic information	Line 88
	5d	Relevant past interventions with outcomes	Line 83
**Clinical Findings**	6	Describe significant physical examination (PE) and important clinical findings.	Line 84-89
**Timeline**	7	Historical and current information from this episode of care organized as a timeline	Line 77-79
**Diagnostic Assessment**	8a	Diagnostic testing (such as PE, laboratory testing, imaging, surveys).	Line 84-95
	8b	Diagnostic challenges (such as access to testing, financial, or cultural)	Line 92
	8c	Diagnosis (including other diagnoses considered)	Line 85 -95
	8d	Prognosis (such as staging in oncology) where applicable	None
**Therapeutic Intervention**	9a	Types of therapeutic intervention (such as pharmacologic, surgical, preventive, self-care)	Line 109
	9b	Administration of therapeutic intervention (such as dosage, strength, duration)	Line 138-141
	9c	Changes in therapeutic intervention (with rationale)	None
**Follow-up and Outcomes**	10a	Clinician and patient-assessed outcomes (if available)	Line 147-149
	10b	Important follow-up diagnostic and other test results	None , .limitation part
	10c	Intervention adherence and tolerability (How was this assessed?)	Line208-211
	10d	Adverse and unanticipated events	None
**Discussion**	11a	A scientific discussion of the strengths AND limitations associated with this case report	Line 213-220 / 298-305
	11b	Discussion of the relevant medical literature **with references**.	Line 224-230 / Line286-288
	11c	The scientific rationale for any conclusions (including assessment of possible causes)	Line281-291
	11d	The primary "take-away" lessons of this case report (without references) in a one paragraph conclusion	Line 306-311
**Patient Perspective**	12	The patient should share their perspective in one to two paragraphs on the treatment(s) they received	Line 147-149
**Informed Consent**	13	Did the patient give informed consent? Please provide if requested	Yes ☑ No ☐
